# A 3D DC Electric Field Meter Based on Sensor Chips Packaged Using a Highly Sensitive Scheme

**DOI:** 10.3390/mi16040484

**Published:** 2025-04-20

**Authors:** Pengfei Yang, Xiaolong Wen, Xiaonan Li, Zhaozhi Chu, Chunrong Peng, Shuang Wu

**Affiliations:** 1 Beijing Key Laboratory for Sensors, School of Applied Science, Beijing Information Science and Technology University, Beijing 100192, China; pfy@bistu.edu.cn (P.Y.); lixiaonan824@163.com (X.L.); 2 School of Mathematics and Physics, University of Science and Technology Beijing, Beijing 100083, China; xiaolongwen@ustb.edu.cn; 3 Institute of Microelectronics of Chinese Academy of Sciences, Beijing 100029, China; 4 State Key Laboratory of Transducer Technology, Aerospace Information Research Institute, Chinese Academy of Sciences, Beijing 100094, China; crpeng@mail.ie.ac.cn; 5 Beijing Tflying Transducer Technology Co., Ltd., Beijing 100083, China; wushuang@tflying.cn

**Keywords:** MEMS, 3D electric field meter, sensor chips, moistureproof packaging structure, highly sensitive package

## Abstract

This study presents a 3D DC electric field meter (EFM) that uses three identical 1D MEMS chips. The shielding electrodes and sensing electrodes of the MEMS chips employ a combination of rigid frames and short strip-type beams to improve vibrational stability. To enhance sensitivity, our MEMS chips feature inner convex packaging covers. Moreover, the integrated design and wireless transmission efficiently eradicate the impact of ground potential on detection results. Detailed simulations have been conducted to analyze the electric field distribution within the chip package and the electric field distribution on the EFM’s surface. A prototype was then developed, calibrated, and validated. The test results indicate that the sensitivity of our proposed 3D EFM is at least 4.64 times higher than the highest sensitivity observed in previously reported MEMS 3D EFMs. The maximum relative deviation is a mere 2.2% for any rotation attitude. Remarkably, even in high humidity conditions, the EFM’s linearity remains within 1%. Additionally, the resolution of any single axis is less than 10 V/m.

## 1. Introduction

Electric field measurement plays a pivotal role in both scientific research and engineering technologies. For example, in the realm of meteorology, the atmospheric electric fields are monitored for lightning hazard warning and climate change assessment [1,2]. In power systems, measuring electric fields in close proximity to high-voltage equipment is vital. Such measurements ensure the safety of personnel and equipment during live-line maintenance [3] and secure avoidance of power lines by helicopters [4]. They also allow for credible electromagnetic environment assessments [5]. Furthermore, in industrial production lines, electrostatic voltage is a critical factor, obtained via DC electric field measurement. Not only does it provide a direct reading of the electrostatic charge present in products, but it also offers undeniable evidence when industrial static elimination systems fail [6].

Currently, most electric field meters (EFMs) are only capable of measuring one-dimensional (1D) electric fields [7,8]. While they are sufficient for the application needs of electric field detection in many scenarios, there are still scenarios where they fall short in accurately detecting unknown electric fields. For instance, at high altitudes, apart from the electric field component perpendicular to the ground, a crucial horizontal electric field component parallel to the ground is present, intensifying with increasing altitude. This horizontal component offers valuable information about the direction of thundercloud movement [9,10]. Additionally, buildings surrounding high-voltage DC transmission lines often significantly distort the electric field, making it challenging to determine its direction [11]. If a single-axis EFM is utilized for measuring the distorted electric field of a high-voltage DC line, obtaining a comprehensive and accurate depiction of the field’s spatial distribution becomes significantly challenging. As a result, it is essential to pursue the development of a three-dimensional (3D) electric field detection instrument to complement current electric field detection methods.

A variety of techniques have been proposed for measuring the electric field vector, with the field mill being a commonly applied technical method. Predominantly, there are two types of 3D EFMs based on the field mill principle: spherical EFMs [12] and hybrid EFMs [11]. Nevertheless, these field-milled 3D EFMs encounter substantial mechanical wear and power consumption issues. Moreover, despite reports of passive 3D EFMs [13] and integrated optical 3D EFMs [14], they are not fit for DC electric field measurements due to the limitations inherent in their working principles.

Over the past decade, studies have focused on microelectromechanical system (MEMS)-based 3D EFMs due to their notable advantages in wear prevention, reduced power consumption, and compact size. In 2014, Wen et al. proposed a coplanar MEMS 3D EFM, utilizing identical 1D electric field sensor chips (EFSCs) arranged arbitrarily on the same plane for 3D electric field detection [15]. Fast forward to 2016, Li et al. introduced a decoupling calibration method to suppress cross-axis coupling interference, employing a genetic algorithm for the orthogonally assembled 3D EFM [16]. A year later, Ling et al. reported a single-chip 3D electric field microsensor with an in-plane rotary mechanism for the simultaneous detection of the electrostatic field components along the *X*-, *Y*-, and *Z*-axes [17]. By 2018, Ling et al. designed a 1D EFSC presenting a low cross-axis coupling interference and used this 1D EFSC to develop a 3D EFM [18]. Fast forward to last year, 2023, Peng et al. proposed a single-chip 3D electric field microsensor with piezoelectric excitation to minimize driving voltage and feedthrough [19]. Although the issue of cross-axis interference in the aforementioned MEMS 3D field meters has been addressed, the influence of environmental humidity on measurement accuracy remains unconsidered. For packaged EFSCs, measurement accuracy deteriorates when the environmental relative humidity exceeds 60% [1]. In single-chip structures, which lack protective packaging, the impact of ambient humidity on measurement accuracy is even more pronounced. Furthermore, existing MEMS 3D EFMs exhibit low sensitivity; for instance, a maximum uniaxial sensitivity of 3.53 mV/kV/m has been reported [16], which falls short of the requirements for high-sensitivity detection. Additionally, EFSCs typically use elongated strip-type beams, which may cause potential operational stability issues. This study aims to improve the sensitivity and stability of MEMS 3D EFMs, as well as addressing the challenge posed by environmental humidity on measurement accuracy.

This paper proposes a more pragmatic 3D DC EFM utilizing three identical 1D EFSCs, featuring the following enhancements:

1. A moistureproof packaging structure has been incorporated into the design of the new 3D field meter to counter the effects of ambient humidity on the measurement’s accuracy.
2. The sensing units of the EFSCs use a combination of hard frames and short strip-type beams to improve vibration stability.
3. Contrary to the flat packaging covers on EFSCs reported previously, the EFSCs in the proposed structure use inward-convex packaging covers, further improving the sensitivity of the 3D field meter.
4. The proposed solution necessitates only the detecting electrodes to be assembled orthogonally, with no restrictions on the arrangement of EFSCs—a welcome flexibility for the EFM’s implementation.
5. Unlike 3D EFMs in previous papers which contained probes separated from their signal conditioning circuit, and conveyed signals through wires, the proposed 3D field meter has been designed as an integrated structure. Here the data are transmitted wirelessly, eradicating potential issues of detection results being affected by the ground potential due to changes in the installation location of the signal conditioning circuit.

## 2. Microsensor and Its Package

**Figure 1:**
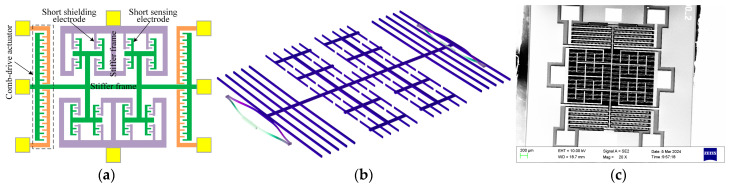
Improved 1D EFSC. (**a**) Schematic diagram. (**b**) Lateral vibration mode, resonant frequency of 3012 Hz. (**c**) SEM image.

The microsensor primarily consists of an electrostatically driven resonator, which detects the incident electric field through lateral vibration modulation [20]. In the previously reported microsensor structures, elongated strip-type beams served as the shielding and sensing electrodes. However, the swing of these long beams presented potential operational stability issues during vibration and could even cause absorption in the device structure. To address the possible vibration instability problems, the shielding electrodes and sensing electrodes of the 1D EFSCs used in this study were redesigned, as shown in Figure 1a. The shielding electrodes of the new sensor chip are structured into a stiffer frame from which short strip-type beams extend. Similarly, short sensing electrodes are also installed on the fixed frame structure. Two arrays of sensing electrodes are symmetrically positioned and coupled to their coplanar shielding electrodes. This new design significantly enhances the stability of the resonator vibration compared to the previously reported microsensor structures.

To achieve a large vibration amplitude with low driving voltages, the sensor chip is designed to operate at its resonant frequency. According to the operational principle, the sensor chip requires the lateral vibration mode, as illustrated in Figure 1b. The lateral vibration resonance frequency of the upgraded microsensor is 3012 Hz. The microsensors were fabricated using a silicon-on-insulator process. Figure 1c depicts the scanning electron microscope (SEM) photograph of the fabricated microsensor, and its size is approximately 4.5 mm × 4.5 mm. The new microsensor is driven by an 18 V common DC bias voltage and antisymmetric 1.6 V_p-p_ sinusoidal voltages applied on either side of the comb-drive actuator. It can detect electric fields exceeding 600 kV/m.

**Figure 2:**
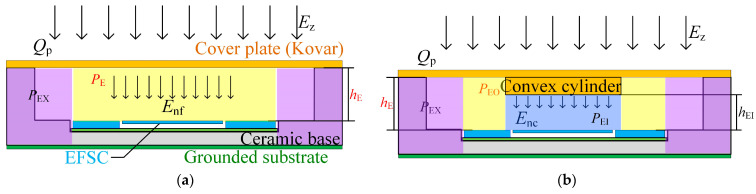
Schematic of the EFSC package. (**a**) Flat cover plate. (**b**) Inward-convex cover plate.

The sensitivity of the EFSC is closely tied to its packaging structure. Yang et al. introduced the microsensor’s packaging solution based on a ceramic base featuring a flat Kovar cover plate, as depicted in Figure 2a [21]. This sensor chip enables the detection of the incident electric field by measuring the electric field between the cover plate and the ceramic substrate. However, given that the 3D electric field detection employs a floating-ground method, the sensitivity of the reported microsensor after packaging falls short of actual use requirements. Hence, we proposed a highly sensitive packaging structure furnished with an inward-convex cover to enhance the EFSC’s detection sensitivity. Figure 2b shows the schematic of the newly designed package.

The EFSC package with a flat cover plate can be divided into two regions: $P_{E}$ (yellow-colored region) and $P_{EX}$ (purple-colored regions), as demonstrated in Figure 2a. The capacitances in the $P_{E}$ and $P_{EX}$ areas are represented as $C_{PE}$ and $C_{PEX}$, respectively. Here $C_{PE}+C_{PEX}=C_{p}$, $C_{p}$ is the capacitance of the EFSC package. A summary of the capacitance definition for different regions of the EFSC package is listed in Table 1.

Additionally, $C_{PE}$ can be equivalent to the ideal parallel-plate capacitance without taking into account the fringing effects. Consequently, the voltage $V_{pf}$ on the flat packaging cover plate is expressed as

(1) $$V_{pf}=\frac{-Q_{p}}{\frac{\varepsilon A_{PE}}{h_{E}}+C_{PEX}}$$

where $Q_{p}$ is the amount of charge generated on the packaging cover plate under the incident electric field, $A_{PE}$ is the area of the $P_{E}$ region when viewed from above, ε is the permittivity of the air inside the package, and $h_{E}$ is the distance between the packaging cover plate and the microsensor.

The voltage $V_{pf}$ produces an electric field $E_{nf}$ that is perpendicular to the microsensor inside the ceramic package. Because the distribution of the electric field between the flat packaging cover plate and the sensing unit of the microsensor is uniform enough [22], $E_{nf}$ can be solved by

(2) $$E_{nf}=\frac{V_{pf}}{h_{E}}=\frac{-Q_{p}}{\varepsilon A_{PE}+h_{E}C_{PEX}}$$

As illustrated in Figure 2b, the high-sensitivity package’s cover plate protrudes a cylinder inwards. This design contrasts with the package structure seen in Figure 2a. The $P_{E}$ region in Figure 2b can be also split into two parts: $P_{EO}$ (yellow-colored region) and $P_{EI}$ (light blue-colored region). The capacitances in the $P_{EO}$ and $P_{EI}$ regions are denoted by $C_{PEO}$ and $C_{PEI}$, respectively. Here, $C_{PEO}+C_{PEI}=C_{PE}$. Neglecting fringing effects, the voltage $V_{pc}$ on the inward-convex packaging cover plate is expressed as

(3) $$V_{pc}=\frac{-Q_{p}}{\frac{\varepsilon A_{PEO}}{h_{E}}+\frac{\varepsilon A_{PEI}}{h_{EI}}+C_{PEX}}$$

where $A_{PEO}$ and $A_{PEI}$ represent the areas of the $P_{EO}$ and $P_{EI}$ regions on the top view direction, respectively, and $h_{EI}$ is the distance between the convex cylinder of the packaging cover plate and the microsensor ($0<h_{EI}<h_{E}$).

Because of the uniform distribution of the electric field $E_{nc}$ between the inner convex cylinder of the packaging cover plate and the microsensor, as illustrated in Section 4.1, $E_{nc}$ can be derived as

(4) $$E_{nc}=\frac{-Q_{p}}{\varepsilon \left(A_{PEO}\frac{h_{EI}}{h_{E}}+A_{PEI}\right)+h_{EI}C_{PEX}}$$

Taking into account that $A_{PEI}=A_{PE}-A_{PEO}$, Equation (4) can be further modified as

(5) $$E_{nc}=\frac{-Q_{p}}{\varepsilon \left[{A_{PE}-A}_{PEO}\left(1-\frac{h_{EI}}{h_{E}}\right)\right]+h_{EI}C_{PEX}}$$

By comparing Equations (5) and (2), it can be found that $\left|E_{nc}\right|>\left|E_{nf}\right|$ as a result of $0<A_{PEO}\;\left(1-h_{EI}/h_{E}\right)<A_{PE}$, and $h_{EI}<h_{E}$. This conclusion indicates that under the same condition of the measured electric field $E_{z}$, the electric field that exists over the sensor chip inside the protruding cover plate package is greater than that inside the flat cover plate package. Thus, this newly designed packaging structure exhibits higher sensitivity compared to the previously reported flat cover plate package. Moreover, as can be seen from Equation (5), we could further enhance the sensitivity of the new package by expanding $A_{PEO}$ or reducing $h_{EI}$. Nevertheless, the $A_{PEO}$ is limited to a certain range because $A_{PEI}$ must cover the electric field sensing unit of the sensor chip.

**Figure 3:**
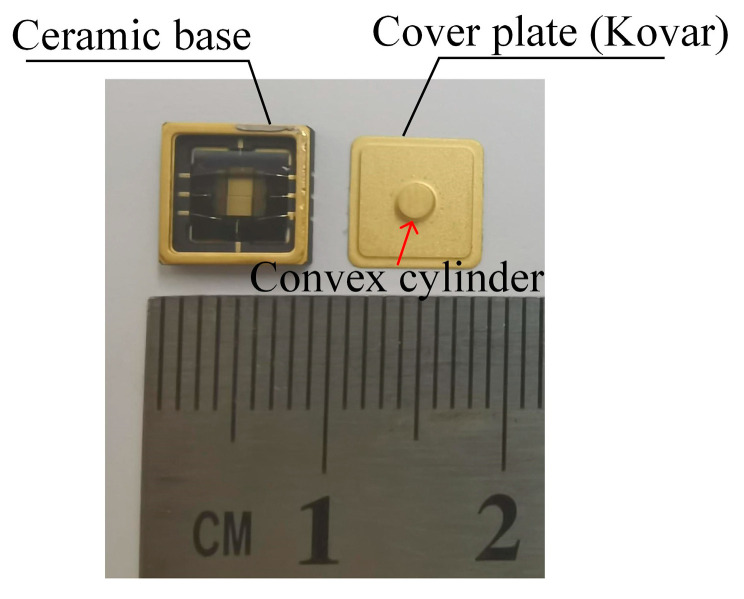
Photograph of the highly sensitive package.

The fabricated high-sensitivity package is presented in Figure 3. The package features an inner convex cylinder with a diameter of 2 mm and a height of 0.78 mm. The distance $h_{E}$ is 0.92 mm, and $h_{EI}$ is 0.14 mm. The area $A_{PE}$ of the $P_{E}$ region is $2.025\times 10^{-5}\;m^{2}$. $A_{PEO}$ is $1.711\times 10^{-5}\;m^{2}$. The overall measured dimensions of the entire package are approximately 7.9 mm × 7.9 mm × 2.2 mm.

**Figure 4:**
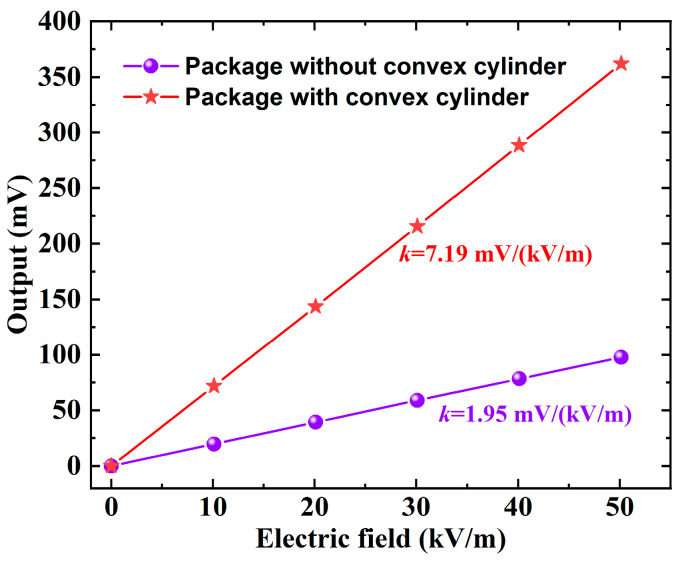
Sensitivity test results of the EFSC with different packaging schemes.

The sensitivity test results of the packaged microsensors are shown in Figure 4. The sensitivity of the EFSC with a flat cover package is 1.95 mV/(kV/m), while the sensitivity of the EFSC with an inner convex cover package is 7.19 mV/(kV/m), which is 3.68 times higher (in the absence of considering the variations among the microsensors). These results further validate the accuracy of the theoretical analysis.

## 3. Vector Fieldmeter

### 3.1. Anti-Humidity Scheme and Structural Arrangement

**Figure 5:**
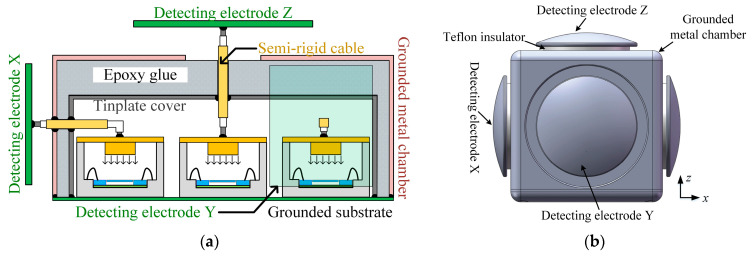
(**a**) Simplified schematic of the proposed 3D DC EFM and (**b**) its arrangement.

Initial experiments have exposed that in high-humidity environments, the ceramic base of the microsensor package tends to polarize readily under a strong incident field, leading to electric field attenuation inside the package and the zero drift of the EFSC [22]. Thus, it substantiates the need for the highly sensitive packages, such as the one proposed in this study, to be protected against moisture. In the literature [1], our team reported on a moisture-resistant packaging structure for 1D electric field detection. Both indoor environmental experiments and outdoor tests demonstrated that this solution yielded favorable results in moisture resistance. Based on our prior research, a new moistureproof packaging structure for 3D electric field detection is proposed, as depicted in Figure 5a. It consists of a grounded substrate, packaged EFSCs, a tinplate cover, a grounded metal chamber, semi-rigid cables, detecting electrodes, and cured epoxy adhesive. Here, semi-rigid cables are coaxial cables with a solid metal outer conductor, typically made of copper or aluminum, that maintains a fixed shape once bent. For achieving 3D electric field detection, a minimum of three packaged EFSCs are mounted on a grounded substrate. Meanwhile, a tinplate cover is sufficient for the protection of the packaged EFSCs. The detecting electrodes are orthogonally arranged for measuring the three components of the incident electric field. The detecting electrodes for the *X*-axis, *Y*-axis, and *Z*-axis are correspondingly connected electrically to the packaging cover plates of the EFSCs via semi-rigid cables in sequence.

Figure 5b illustrates the design layout of the proposed 3D DC EFM. The detecting electrodes, fixed on a cube-shaped grounded metal chamber, are kept separate from the grounded metal chamber by Teflon insulators. Aiming to maintain symmetry in the EFM structure and minimize measurement discrepancies due to structural asymmetry, five detecting electrodes are arranged on the five surfaces of the cube, excluding the bottom. Of these, only three are electrically connected to the package covers of the EFSCs, in a mutually orthogonal arrangement, while the remaining two electrodes are left floating. A noteworthy point from the schematic design and structural configuration discussed above is that the proposed EFM necessitates only three detecting electrodes assembled perpendicularly to each other. However, there exist no constraints concerning the arrangement of the EFSCs. This flexibility greatly benefits the EFM instrument implementation. Since the EFSCs are not required to be oriented orthogonally, the associated interface circuits can be more easily designed, and the internal structure of the 3D EFM can be made more compact and space-efficient.

### 3.2. Measurement Principle

**Figure 6:**
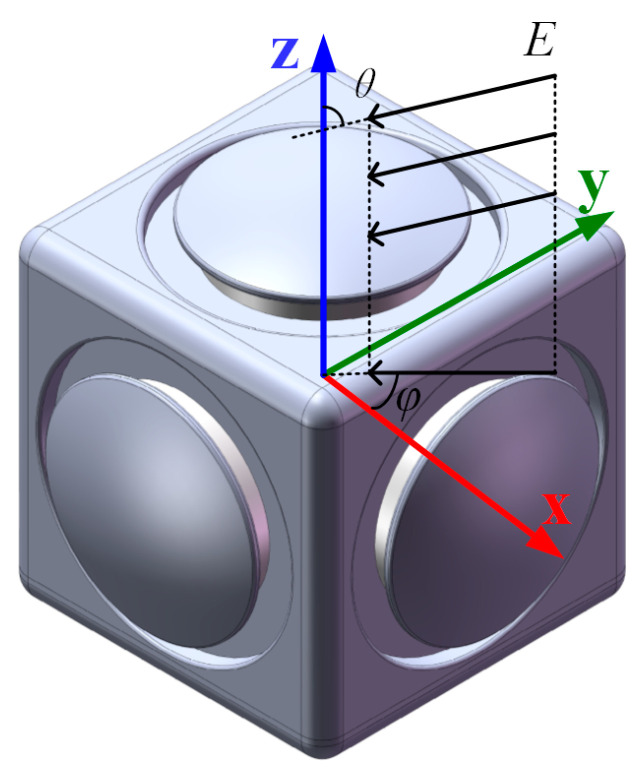
Conceptual drawing of the position of the local coordinate system.

A local coordinate system is set up, using the center of the 3D EFM as its point of origin, as shown in Figure 6. Here, the *X*- and *Y*-axes are in horizontal directions perpendicular to each other, whereas the *Z*-axis points vertically. These *X*-, *Y*-, and *Z*-axes are perpendicular to the detecting electrodes in various directions. The angle between the incident electric field E and the *Z*-axis is identified as θ, while φ is the angle between the horizontal projection of E and the *X*-axis. Considering that the three sensing axes of the proposed EFM measure the electric field in an identical manner, we focus on analyzing one of these sensing axes, referred to here as the *i*-axis, i=X,Y,Z. The output voltage $v_{i}$ of the *i*-axis electric field measurement unit can be given by [1].

(6) $$v_{i}\left(t\right)=-\frac{1}{2}\alpha_{i}\beta R_{f}G_{\mathrm{INA}}V_{a}E_{i}\left(t\right)$$

where β is the conversion gain of the microsensor, $R_{f}$ is the feedback resistance for the microsensor’s analog front end (AFE), $G_{\mathrm{INA}}$ is the gain of the AFE’s instrumentation amplifier, $V_{a}$ is the amplitude of the microsensor’s sinusoidal drive signal, and $E_{i}$ is the electric field distributed vertically on the *i*-axis detecting electrode.

The ratio $\alpha_{i}$ of the electric field $E_{nci}$ inside the package to $E_{i}$ is defined as

(7) $$\alpha_{i}=\frac{\varepsilon A_{di}}{\left(C_{mi}+C_{si}+C_{p}\right)h_{EI}}$$

where $A_{di}$ is the effective area of the *i*-axis detecting electrode, $C_{mi}$ is the capacitance between the *i*-axis detecting electrode and the metal chamber, and $C_{si}$ is the semi-rigid cable capacitance for the *i*-axis.

As depicted in Figure 6, when the 3D EFM is situated in a uniform electric field E, the nearby electric field around the EFM is distorted, and the charge induced on different detecting electrodes relies on the direction of E. Unfortunately, due to the distortion effect of the electric field, the *X*-axis detection electrode measures not just the *X* component of the evaluated electric field $E_{x}$ but also experiences cross-axis coupling interferences from the *Y* and *Z* components of the evaluated electric field components $E_{y}$ and $E_{z}$. Hence, the output voltage $v_{x}$ from the *X*-axis electric field sensing unit can be expressed as

(8) $$v_{x}=k_{xx}E_{x}+k_{xy}E_{y}+k_{xz}E_{z}$$

Similarly, the outputs $v_{y}$ and $v_{z}$, which originate from the electric field sensing units on the *Y*-axis and *Z*-axis, can be written, respectively, as

(9) $$v_{y}=k_{yx}E_{x}+k_{yy}E_{y}+k_{yz}E_{z}$$

(10) $$v_{z}=k_{zx}E_{x}+k_{zy}E_{y}+k_{zz}E_{z}$$

where $k_{mn}$ is the sensitivity coefficient of the electric field component $E_{n}$ at the *m*-axis detecting electrode, m=x,y,z, n=x,y,z; when m=n, $k_{mn}$ is known as the coaxial sensitivity, but when m≠n, $k_{mn}$ is dubbed the coupling sensitivity. Equations (8)–(10) can be expressed by the following matrix:

(11) $$\left[\begin{array}{c} v_{x}\\ v_{y}\\ v_{z} \end{array}\right]=\left[\begin{array}{ccc} k_{xx} & k_{xy} & k_{xz}\\ k_{yx} & k_{yy} & k_{yz}\\ k_{zx} & k_{zy} & k_{zz} \end{array}\right]\left[\begin{array}{c} E_{x}\\ E_{y}\\ E_{z} \end{array}\right]$$

Solving Equation (11), the three components of the measured electric field are given by

(12) $$\left[\begin{array}{c} E_{x}\\ E_{y}\\ E_{z} \end{array}\right]=C\left[\begin{array}{c} v_{x}\\ v_{y}\\ v_{z} \end{array}\right]$$

where the decoupling matrix C is defined as

(13) $$C={\left[\begin{array}{ccc} k_{xx} & k_{xy} & k_{xz}\\ k_{yx} & k_{yy} & k_{yz}\\ k_{zx} & k_{zy} & k_{zz} \end{array}\right]}^{-1}$$

Finally, $\hat{E}$, which is the amplitude estimation of the measured electric field E, can be determined by

(14) $$\hat{E}=\sqrt{E_{x}^{2}+E_{y}^{2}+E_{z}^{2}}$$

## 4. Numerical Simulation

### 4.1. Simulation of Highly Sensitive Package

**Figure 7:**
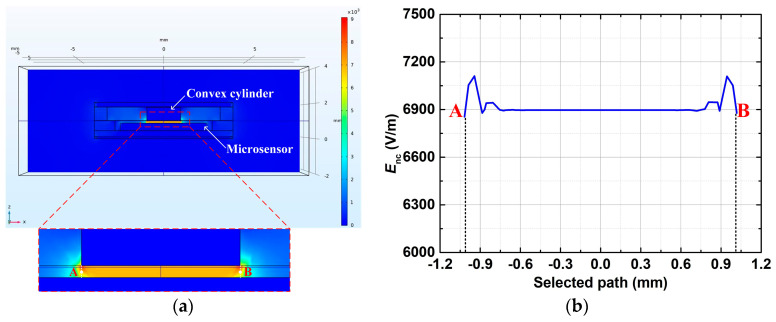
Simulation results of the electric field inside the package: (**a**) cloud map; (**b**) the field change curve from points **A** to **B** at the middle position between the inner convex cylinder and the microsensor.

A numerical simulation model of the package was created using the FEM tool COMSOL Multiphysics (Version 5.4) to validate the uniform distribution of the electric field between the microsensor and the inner convex cylinder of its highly sensitive package. The highly sensitive package is placed in air, which has a relative dielectric constant of 1. The gap $h_{EI}$ between the inner convex cylinder and the microsensor is set at 0.14 mm. The package cover is applied with a voltage of 1 V, while the microsensor is grounded equivalently. Figure 7a presents the simulated results of the electric field cloud map. The curve of the electric field change from point **A**, on one side of the inner convex cylinder, to point **B**, on the opposing side, is shown in Figure 7b. Both points **A** and **B** are located in the middle position between the inner convex cylinder and the microsensor. It can be found from Figure 7b that an unchanging electric field value of 6896.55 V/m prevails over a circular realm of a 0.75 mm radius above the microsensor. However, inside the belt range with a radius extending from 0.75 mm to 1 mm, the electric field value experiences minor fluctuations—a maximum deviation of 214.68 V/m—due to the distortion effect at the boundary of the inner convex cylinder. Despite these fluctuations near the external vicinity of the inner convex cylinder, the observed relative change in the electric field is merely 3%, indicating a fairly uniform electric field distribution between the inner convex cylinder of the package and the microsensor. Additionally, a capacitance value of $C_{p}=1.87\;pF$ for the EFSC’s package is obtained from the simulation.

### 4.2. Simulation of 3D EFM Structure

The 3D EFM structure proposed was analyzed using the same FEM tool from the package simulation to investigate the cross-axis coupling interference and the distribution characteristics of induced charge on the various axes’ detecting electrodes when the EFM is rotated. A cubic space model of 2000 mm side length, capable of generating a uniform electric field, was constructed. The cubic space’s medium was set to air with a relative permittivity of 1. The EFM model was centrally positioned within this cubic space, with the *X*-, *Y*-, and *Z*-axes of the cubic space placed perpendicular to the *X*-axis, *Y*-axis and *Z*-axis detecting electrodes of the EFM, respectively. Several boundary conditions were imposed on the EFM. They include setting the total charge of each axis’s detection electrode to be 0 C, the relative permittivity of the Teflon insulators to be 2.55, and the voltage applied to the grounded metal chamber to be 0 V. The cube space’s top and bottom voltages were set at 1 kV and −1 kV, respectively, resulting in a vertical incident field with an intensity of 1 kV/m directed in the *Z*-axis.

**Figure 8:**
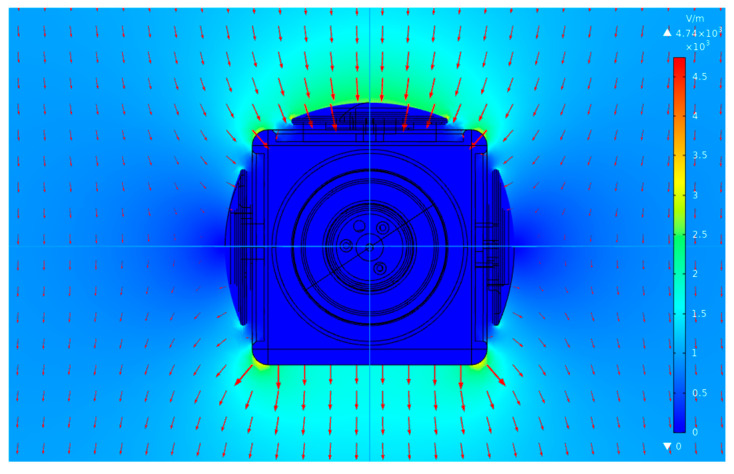
Cloud map and vector map of the 3D EFM. The arrows represent the direction of the electric field.

Figure 8 shows a simulation of the electric field distribution for the 3D EFM. The results highlight a pronounced concentration of electric field lines on the *Z*-axis detecting electrode. With an incident electric field of 1 kV/m along the *Z*-axis, the maximum field around the *Z*-axis detecting electrode is found to be 2.6 kV/m, and the charge is found to be $-3.05\times 10^{-11}\;C$. Considering the EFM structure’s incomplete symmetry, the *X*-axis and *Y*-axis detecting electrodes are seen to induce charges of $2.99\times 10^{-13}\;C$ and $2.84\times 10^{-13}\;C$ respectively. Suppose that the *X*-, *Y*-, and *Z*-axis detecting electrodes of the EFM have an equal ability to induce charges, then the coupling interference ratios of the electric field in the *Z*-axis direction on the *X*- and *Y*-axis detecting electrodes are 0.98% and 0.93%, respectively. This finding further underscores the importance of establishing matrix Equation (11).

**Figure 9:**
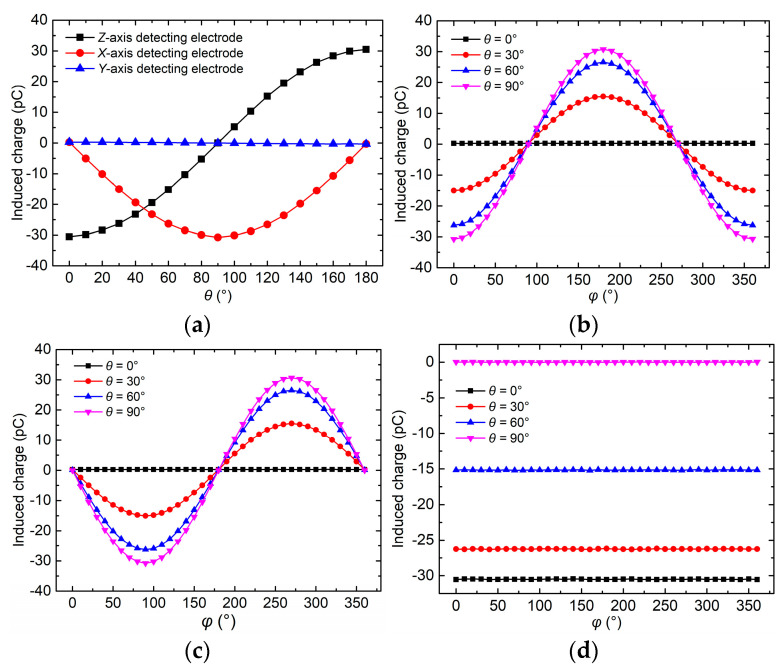
Simulation results of the 3D EFM’s response to the incident electric field with different directions. (**a**) Induced charge on each detecting electrode versus θ when $\varphi =0^{{}^{\circ}}$; (**b**) induced charge on *X*-axis detecting electrode versus φ when $\theta =0^{{}^{\circ}}$, $30^{{}^{\circ}}$, $60^{{}^{\circ}}$, and $90^{{}^{\circ}}$; (**c**) induced charge on *Y*-axis detecting electrode; (**d**) induced charge on *Z*-axis detecting electrode.

Figure 9 provides simulated results of the induced charge on the detecting electrodes of different axes when the 3D EFM undergoes rotation. Figure 9a illustrates that with $\varphi =0^{{}^{\circ}}$, the curves concerning the induced charge on the *X*-axis detecting electrode and the *Z*-axis detecting electrode exhibit strong sinusoidal characteristics with a 90° phase difference. This behavior arises because the rotation of the 3D EFM causes the angle between each detecting electrode’s normal vector and the electric field direction to vary periodically. The observed 90° phase difference results from the fact that the normal vectors of adjacent detecting electrodes are oriented 90° apart in space, making their electric field projections orthogonal and thus resulting in phase-shifted sinusoidal responses. On the other hand, Figure 9b–d show that when $\theta \neq 0^{{}^{\circ}}$, the curves corresponding to the induced charge on the *X*-axis detection electrode and the *Y*-axis detection electrode exhibit prominent sinusoidal characteristics, again maintaining a 90° phase difference. However, the *Z*-axis detection electrode’s induced charge stays constant.

## 5. Implementation

### 5.1. Proposed Structure

**Figure 10:**
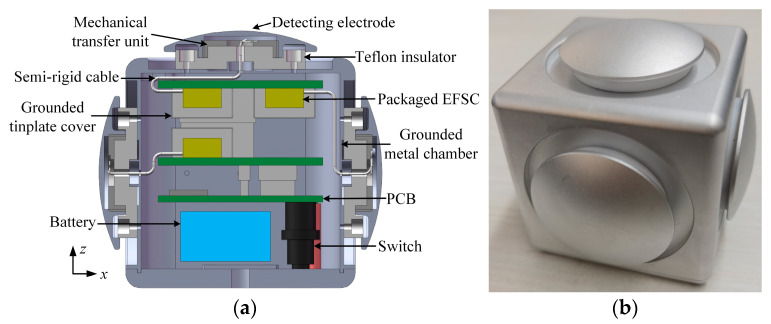
Proposed 3D EFM device. (**a**) Cross section shows instrument arrangements; (**b**) prototype.

Figure 10a provides a look at the main features of the 3D EFM prototype. Teflon insulators and mechanical transfer units are mounted on the grounded metal chamber via screws in sequence. Printed circuit boards (PCBs) with the packaged EFSCs protected by tinplate covers are placed inside the metal chamber. One end of the semi-rigid cable extends through the metal chamber, and its core is electrically connected to the mechanical transfer unit using screws. The detecting electrodes then get mounted onto these mechanical transfer units through internal thread connections. The detecting electrodes, mechanical transfer units, and metal chamber are all made of aluminum. The battery is also installed inside the metal chamber, and the switch controls the startup of the EFM. Figure 10b provides a snapshot of the manufactured EFM prototype. The physical dimensions of the EFM prototype are 74 mm×74 mm×67 mm.

Since all three measurement units of the manufactured 3D EFM have precisely the same structure, the capacitance $C_{mi}$ between the *i*-axis detecting electrode and the metal chamber is calculated to be 24.31 pF, and the semi-rigid cable capacitance $C_{si}=1.96\;pF$ for all electric field measurement units. The capacitance of the EFSC package is $C_{p}=1.87\;pF$, and the distance between the inner convex cylinder of the package and the microsensor is $h_{EI}=0.14\;mm$. Referencing the results of the simulation detailed in Section 4.2, the induced charge on the *i*-axis detecting electrode is $-3.05\times 10^{-11}\;C$ under a vertical incident field $E_{i}=1\;kV/m$. Applying these values into Equation (7), we compute the ratio $\alpha_{i}$ to be 7.48. However, neglecting humidity influences, if the EFM merely includes highly sensitive packaged EFSCs without external detecting electrodes, the ratio $\alpha_{i}$ reduces to 2.11. Therefore, the proposed structure accomplishes two vital goals: it mitigates the effect of environmental humidity, and it enhances detection sensitivity.

### 5.2. Interface Circuit

**Figure 11:**
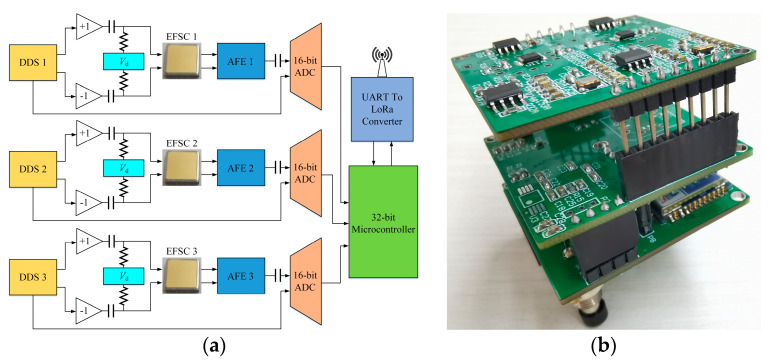
Proposed 3D EFM circuitry. (**a**) Schematic view; (**b**) photograph.

Figure 11 shows the circuitry of the 3D EFM. Considering that the three 1D EFSCs are independent of each other and need to be measured simultaneously, the interface circuit is suitably segmented into three identical signal-conditioning channels. This configuration allows for parallel processing and individual readouts from each 1D EFSC. A 16-bit analog-to-digital converter (ADC) digitizes both the output signal of the AFE and the sinusoidal drive voltage. The system uses an embedded 32-bit microcontroller to accomplish synchronous demodulation. To avoid ground potential impact on the measurement, the outputs of the EFM are sent wirelessly from a universal asynchronous receiver–transmitter to an LoRa converter directly to a laptop.

## 6. Experimental Results

### 6.1. Experiment Setup

**Figure 12:**
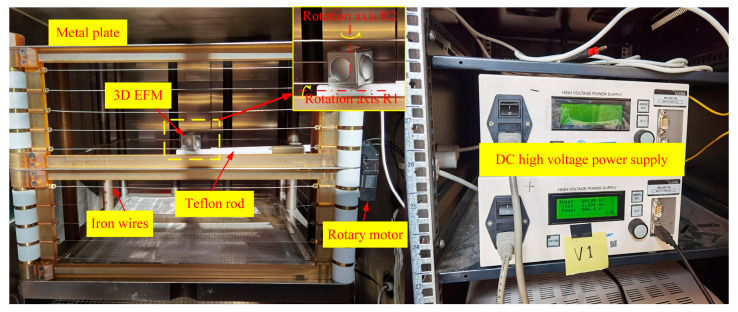
Calibration setup for the proposed 3D EFM.

The 3D EFM prototype was calibrated using a parallel-plate apparatus with a side dimension of 90 cm and a spacing of 60 cm, as depicted in Figure 12. However, for finite-sized metal plates, the actual field between parallel conducting plates was not ideally uniform due to the effects of fringing. To control the fringing errors, IEEE Standard 644 [22] recommends the use of iron wires as grading rings, placed to surround the parallel conducting plates at equal intervals. An Iseg HP 500 power supply served to generate the required DC high voltage. We developed a rod-shaped bracket, made of Teflon, to fix the 3D EFM in the parallel-plate apparatus and control its orientation. The axis of this rod-shaped bracket was parallel to the conducting plates and perpendicular to their opposing sides. One end of the rod-shaped bracket, situated in the center of the parallel-plate apparatus, was designated for mounting the 3D EFM. The other end connected to a rotary motor. The unique design of the bracket incorporated two orthogonal and intersecting rotation axes, R1 and R2, and their intersection coincided with the center of the 3D EFM. This arrangement ensured the 3D EFM’s center remained unchanged during rotation. Prior to testing, the direction of the *Z*-axis detecting electrode of the 3D EFM was parallel to the applied electric field, and the direction of its *X*-axis detecting electrode aligned parallel to the rotation axis R1.

### 6.2. Sensitivity Matrix

**Figure 13:**
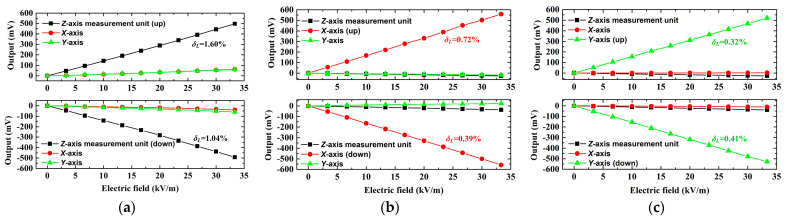
Calibration results. (**a**) The electric field is perpendicular to the *Z*-axis detecting electrode. (**b**) The electric field is perpendicular to the *X*-axis detecting electrode. (**c**) The electric field is perpendicular to the *Y*-axis detecting electrode.

The 3D EFM underwent uniaxial calibration to determine the sensitivity $k_{mn}$. The applied voltage to the parallel-plate apparatus was varied from 0 to 20 kV in 2 kV increments, which generated a standard electric field ranging from 0 to 33.33 kV/m, stepped at 3.33 kV/m intervals. The *Z*-axis measurement unit of the 3D EFM was first calibrated. To enhance calibration accuracy, after testing the *Z*-axis measurement unit in the upward direction, rotate it and then calibrate it in the downward direction. While calibrating the *Z*-axis measurement unit, we simultaneously recorded output data from the *X*- and *Y*-axis measurement units. The calibration curve of the *Z*-axis measurement unit is illustrated in Figure 13a. The sensitivity coefficient $k_{mz}$ is determined by averaging the results from the upward and downward calibrations, followed by fitting a straight line using the least squares method, m=x,y,z. Next, by rotating the 3D EFM and applying the same calibration method as used on the *Z*-axis measurement unit, the *X*- and *Y*-axis measurement units were calibrated, as represented in Figure 13b,c. The linearity $\delta_{L}$ is within a 1.6% range. This demonstrates that the prototype displays robust linearity in response to the incident electric field. Finally, according to Equation (11), the prototype’s sensitivity matrix is given by

(15) $$K=C^{-1}=\left[\begin{array}{ccc} 16.77 & 0.19 & 1.53\\ -0.62 & 15.77 & 1.81\\ 0.17 & 0.18 & 14.78 \end{array}\right]\;\left(mV\cdot {kV}^{-1}\cdot m\right)$$

Besides, the maximum cross-axis sensitivity calculated from Equation (15) is 11.86%.

A uniaxial sensitivity comparison between the proposed scheme and the existing MEMS 3D EFM has been conducted, and the results are listed in Table 2. Owing to the use of highly sensitive packaged EFSCs and the humidity-resistant packaging structure that can improve sensitivity, the sensitivity achieved in this study is at least 4.64 times higher than the highest sensitivity recorded in the previously reported MEMS 3D EFM.

### 6.3. Vector Electric Field Verification Tests

**Figure 14:**
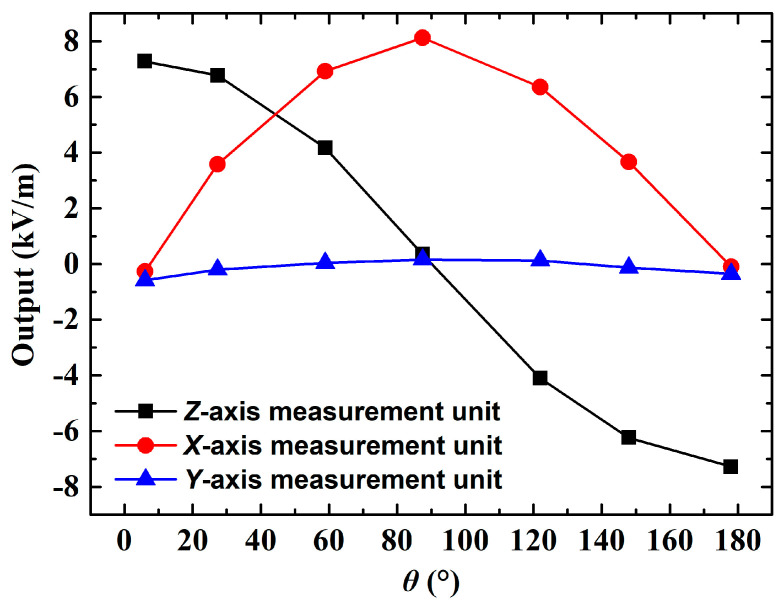
Test results of the 3D EFM rotating around the R1 axis when E=8 kV/m.

When exposed to a uniform electric field of 8 kV/m, the 3D EFM rotates around the R1 axis and the resultant electric field outputs from its three measurement units are depicted in Figure 14. It is obvious that the electric field curves of the *X*- and *Z*-axis measurement units display sinusoidal characteristics and are separated by an approximate phase difference of 90°, which is consistent with the simulation results in Figure 9a. One notable contrast is that since Figure 14 represents electric field curves, they are exactly opposite to the induced charge curves shown in Figure 9a. Additional observations from Figure 14 reveal that the *Y*-axis measurement unit’s output does not consistently hold at 0. This may be attributed to two likely factors. One, adjusting φ to the ideal $0^{{}^{\circ}}$ during testing could be challenging, and two, the Teflon-made rod-shaped bracket may not be robust enough. A slight bend may be introduced once the 3D EFM is installed, impacting the *Y*-axis output.

Verification tests were conducted to evaluate the measurement accuracy of the developed 3D EFM. The parallel-plate apparatus generated a uniform electric field of 10 kV/m. The 3D EFM was randomly rotated into eight different attitudes. For each attitude, the estimated electric field was recorded, and the relative deviation (RD) between this estimate and the original applied electric field was calculated. The data are tabulated and given in Table 3. The maximum RD recorded was a mere 2.2%. The verification test results show that the estimated electric field is mostly consistent with the applied electric field. However, some measurement discrepancies still exist, which could be attributed to the calibration errors, as well as deviations caused by a slight bending of the rod-shaped bracket after installing the 3D EFM.

### 6.4. Moistureproof Verification Tests

**Figure 15:**
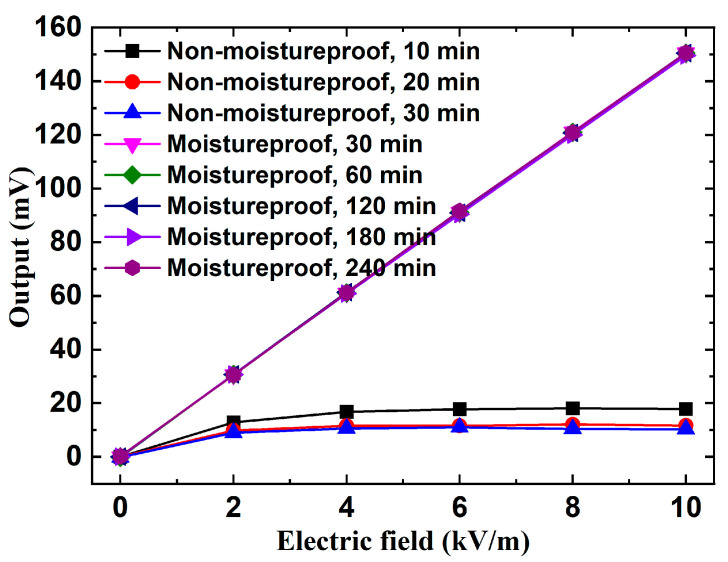
Verification test curves of non-moistureproof and moistureproof 3D EFM at 99% relative humidity for different times.

Verification experiments were carried out within a temperature and humidity test chamber (THTC) to examine the impact of environmental humidity on the sensitivity and linearity of the 3D EFM. A small parallel-plate calibrator with a side length of 30 cm and an inter-plate space of 20 cm was placed in the THTC. The THTC settings were fixed at 99% relative humidity and a temperature of 25 °C. During the tests, it was difficult to rotate the 3D EFM within the THTC. Due to identical electric field detection and moisture prevention schemes in all three measurement units of the 3D EFM, only the *Z*-axis measurement unit was selected for the humidity verification test. The first phase of the experiment involved placing the 3D EFM, untreated for moisture resistance, in the THTC. Calibration tests were sequentially conducted after durations of 10, 20, and 30 min. Subsequently, a moisture-resistant 3D EFM was placed in the THTC. In this instance, calibration tests were performed following periods of 30, 60, 120, 180, and 240 min. The calibration curves from both experiments are exhibited in Figure 15, with sensitivity and linearity details tabulated in Table 4. It can be clearly seen from the test results that, without moisture-resistance treatment, 3D EFM’s uniaxial sensitivity in a 99% relative humidity environment decays from 15.04 $mV\cdot {kV}^{-1}\cdot m$ in a low-humidity environment to 1.77 $mV\cdot {kV}^{-1}\cdot m$ after just 10 min. Sensitivity progressively decreases over time, and linearity deteriorates, rendering the device unable to meet application requirements. Conversely, the moisture-resistant 3D EFM maintains near-constant uniaxial sensitivity at 15.03 $mV\cdot {kV}^{-1}\cdot m$ after four hours in the same 99% high-humidity setting and holds linearity within 1%. These experimental results validate that the proposed 3D EFM can achieve accurate measurements, even in high-humidity environments.

### 6.5. Zero Output Tests and Resolution Analysis

**Figure 16:**
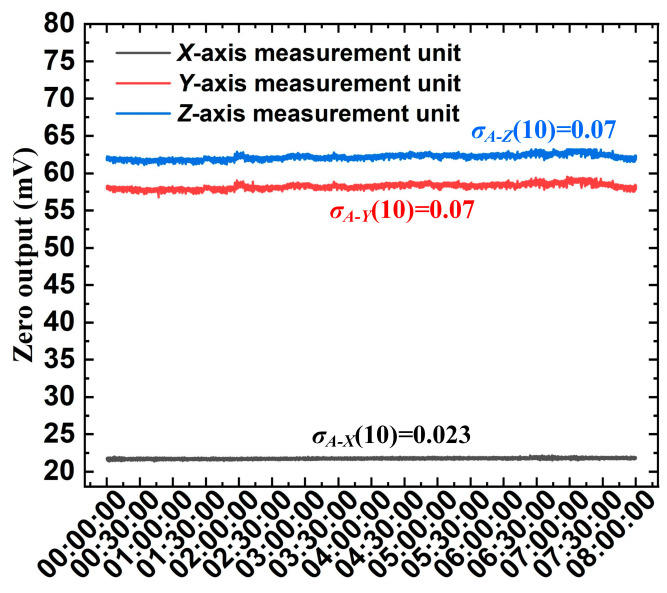
Zero output at a temperature of 20 °C and a relative humidity of less than 20%.

To evaluate the noise characteristics and stability of the 3D EFM, we measured the zero output over 8 h at a temperature of 20 °C and a relative humidity of less than 20% after shielding it in a grounded metal cavity. The data update rate of the 3D EFM is 1 s. The test results are plotted in Figure 16, showing that the output of the 3D EFM exhibits no significant drift. Grouping the data into sets of 10 points, the Allan deviations $\sigma_{A}$ of the zero output of the 3D EFM were calculated as 0.023 (X), 0.07 (Y), and 0.066 (Z), respectively. The resolution R of any sensing axis i of the 3D EFM can be determined by

(16) $$R_{i}=\frac{2\sigma_{A-i}}{k_{ii}}$$

where i=X,Y,Z, Based on the uniaxial sensitivity test results, the resolutions of the *X*-, *Y*-, and *Z*-axis measurement units of the 3D EFM were calculated as 2.7 V/m, 8.9 V/m, and 8.9 V/m, respectively, all of which are below 10 V/m.

## 7. Conclusions

In this paper, a 3D EFM has been developed, and the detailed design, simulation, and implementation method are presented. To solve potential vibration instability issues, the shielding electrodes and fixed sensing electrodes of the 1D EFSCs employed in the EFM were redesigned. We proposed a highly sensitive packaging structure, furnished with an inward-convex cover, to augment the EFSC’s sensitivity further. The integrated design and wireless transmission effectively suppress the impact of ground potential on detection results. It only necessitates that the three detecting electrodes be arranged perpendicularly to each other and imposes no restrictions on the layout of the EFSCs, which is particularly beneficial for the EFM’s implementation. Furthermore, we adopted a humidity-resistant packaging structure in the EFM design, which not only mitigates the effect of environmental humidity but also further improves sensitivity. As a result of the inherent asymmetry of the EFM structure and electric field distortion, a matrix equation was developed to address cross-axis coupling interference. Finite element simulations reveal roughly uniform electric field distribution between the package’s inner convex cylinder and the microsensor. Calibration and verification tests have been carried out. The EFM’s uniaxial sensitivity is at least 4.64 times higher than the highest recorded sensitivity in previously reported MEMS 3D EFMs. The electric field curves of the *X*- and *Z*-axis measurement units exhibit sinusoidal characteristics separated by an approximate phase difference of 90°, consistent with simulation results. The EFM was rotated arbitrarily to eight different attitudes, with the maximum relative deviation recorded being a mere 2.2%. After four hours in high-humidity environments, the EFM maintains near-constant uniaxial sensitivity at 15.03 $mV\cdot {kV}^{-1}\cdot m$ and retains linearity within 1%. Based on the sensitivity test results, the uniaxial resolution of the EFM was found to be less than 10 V/m, as determined by analyzing the zero output.

## Figures and Tables

**Table 1:** Capacitance definition for EFSC package.

Capacitance	Packaging Region
$C_{p}$	The whole package
$C_{PE}$	$P_{E}$, yellow-colored region in Figure 2a
$C_{PEX}$	$P_{EX}$, purple-colored region in Figure 2a
$C_{PEO}$	$P_{EO}$, yellow-colored region in Figure 2b
$C_{PEI}$	$P_{EI}$, light blue-colored region in Figure 2b

**Table 2:** Sensitivity comparison of the proposed scheme with existing MEMS 3D EFM.

MEMS-Based 3D EFM	Year	$\mathrm{Minimum}\;\mathrm{Sensitivity}\;\left(mV\cdot {kV}^{-1}\cdot m\right)$	$\mathrm{Maximum}\;\mathrm{Sensitivity}\;\left(mV\cdot {kV}^{-1}\cdot m\right)$
Assembled 3D EFM with three 1D EFSCs [16]	2016	3.18	3.53
Single-chip 3D EFM based on in-plane rotary mechanism [17]	2017	0.10	0.14
Assembled 3D EFM with low cross-axis interference [18]	2018	0.33	0.46
Microassembled 3D EFM [23]	2019	0.33	1.48
Single-chip 3D EFM with piezoelectric excitation [19]	2023	0.23	2.19
This work	2024	14.78	16.77

**Table 3:** Verification test results with different attitudes.

θ (°)	φ (°)	Estimated Electric Field (kV/m)	RD (%)
174	10	10.03	0.3
135	86	9.85	−1.5
32	88	10.01	0.1
4	51	10.10	1.0
34	277	9.93	−0.7
71	277	9.80	−2.0
122	275	10.22	2.2
165	301	10.01	0.1

**Table 4:** Sensitivities and linearities of non-moistureproof and moistureproof 3D EFM at 99% Relative Humidity for Different Times.

Type	Duration (min)	$\mathrm{Sensitivity}\;k_{zz}$ $\left(mV\cdot {kV}^{-1}\cdot m\right)$	$\mathrm{Linearity}\;\delta_{L}$ (%)
Non-moistureproof	10	1.77	12.32
	20	1.16	19.55
	30	1.04	29.63
Moistureproof	30	15.04	0.32
	60	15.05	0.18
	120	15.04	0.26
	180	14.95	0.77
	240	15.03	0.75

## Data Availability

Data are contained within the article.

## References

[1] Yang P., Wen X., Lv Y., Chu Z., Peng C., Liu Y., Wu S. (2022). Improved microsensor-based fieldmeter for ground-level atmospheric electric field Measurements. IEEE Trans. Instrum. Meas..

[2] Fort A., Mugnaini M., Vignoli V., Rocchi S., Perini F., Monari J. (2011). Design, modeling, and test of a system for atmospheric electric field measurement. IEEE Trans. Instrum. Meas..

[3] Wijeweera G., Bahreyni B., Shafai C., Rajapakse A., Swatek D. (2009). Micromachined electric-field sensor to measure AC and DC fields in power systems. IEEE Trans. Power Delivery..

[4] Williams K.R., De Bruyker D.P.H., Limb S.J., Amendt E.M., Overland D.A. (2014). Vacuum steered-electron electric-field sensor. J. Microelectromech. Syst..

[5] Wang H., Zeng R., Zhuang C., Lyu G., Yu J., Niu B., Li C. (2020). Measuring AC/DC hybrid electric field using an integrated optical electric field sensor. Electr. Power Syst. Res..

[6] Song B., Zhou R., Yang X., Zhang S., Yang N., Fang J., Song F., Zhang G. (2021). Surface electrostatic discharge of charged typical space materials induced by strong electromagnetic interference. J. Phys. D Appl. Phys..

[7] Cui Y., Yuan H., Song X., Zhao L., Liu Y., Lin L. (2018). Model, design, and testing of field mill sensors for measuring electric fields under high-voltage direct-current power lines. IEEE Trans. Ind. Electron..

[8] Harrison R.G., Marlton G.J. (2020). Fair weather electric field meter for atmospheric science platforms. J. Electrost..

[9] Tantisattayakul T., Masugata K., Kitamura I., Kontani K. (2006). Development of the hybrid electric field meter for simultaneous measuring of vertical and horizontal electric fields of the thundercloud. IEEE Trans. Electromagn. Compat..

[10] Xing H., Yang X., Zhang J. (2019). Thunderstorm cloud localization algorithm and performance analysis of a three-dimensional atmospheric electric field apparatus. J. Electr. Eng. Technol..

[11] Liu C., Yuan H., Lv J., Zhao P., Li J., Xu H. (2022). A sensor for 3-D component measurement of synthetic electric field vector in HVDC transmission lines using unidirectional motion. IEEE Trans. Instrum. Meas..

[12] Ravichandran M., Kamra A.K. (1999). Spherical field meter to measure the electric field vector—Measurements in fair weather and inside a dust devil. Rev. Sci. Instrum..

[13] Zhang Z., Li L., Xie X., Xiao D., He W. (2014). Optimization design and research character of the passive electric field sensor. IEEE Sens. J..

[14] Zhang J., Chen F., Sun B., Chen K., Li C. (2014). 3D Integrated optical e-field sensor for lightning electromagnetic impulse measurement. IEEE Photonics Technol. Lett..

[15] Wen X., Fang D., Peng C., Yang P., Zheng F., Xia S. Three dimensional electric field measurement method based on coplanar decoupling structure. Proceedings of the 2014 IEEE SENSORS.

[16] Li B., Peng C., Zheng F., Ling B., Chen B., Xia S. A decoupling calibration method based on genetic algorithm for three dimensional electric field sensor. Proceedings of the 2016 IEEE SENSORS.

[17] Ling B., Wang Y., Peng C., Li B., Chu Z., Li B., Xia S. (2017). Single-chip 3D electric field microsensor. Front. Mech. Eng..

[18] Ling B., Peng C., Ren R., Chu Z., Zhang Z., Lei H., Xia S. (2018). Design, fabrication and characterization of a MEMS-based three-dimensional electric field sensor with low cross-axis coupling interference. Sensors.

[19] Peng S., Zhang Z., Liu X., Gao Y., Zhang W., Xing X., Liu Y., Peng C., Xia S. (2023). A single-chip 3-D electric field microsensor with piezoelectric excitation. IEEE Trans. Electron Devices.

[20] Yang P., Wen X., Chu Z., Ni X., Peng C. (2020). AC/DC fields demodulation methods of resonant electric field microsensor. Micromachines.

[21] Yang P., Wen X., Chu Z., Ni X., Peng C. (2021). Non-intrusive DC voltage measurement based on resonant electric field microsensors. J. Micromech. Microeng..

[22] (2019). IEEE Standard Procedures for Measurement of Power Frequency Electric and Magnetic Fields from AC Power Lines.

[23] Ling B., Peng C., Ren R., Zheng F., Chu Z., Zhang Z., Lei H., Xia S. A microassembled triangular-prism-shape three-dimensional electric field sensor. Proceedings of the 2019 20th International Conference on Solid-State Sensors, Actuators and Microsystems & Eurosensors XXXIII (TRANSDUCERS & EUROSENSORS XXXIII).

